# Efficient evaluation of cellulose digestibility by *Trichoderma reesei* Rut-C30 cultures in online monitored shake flasks

**DOI:** 10.1186/s12934-016-0567-7

**Published:** 2016-09-29

**Authors:** Elena Antonov, Steffen Wirth, Tim Gerlach, Ivan Schlembach, Miriam A. Rosenbaum, Lars Regestein, Jochen Büchs

**Affiliations:** 1 AVT‑Biochemical Engineering, RWTH Aachen University, Worringerweg 1, 52074 Aachen, Germany; 2 Institute of Applied Microbiology, RWTH Aachen University, Worringerweg 1, 52074 Aachen, Germany

**Keywords:** Cellulose, Digestibility, Respiration activity measurement, *Trichoderma reesei* Rut-C30, Beech wood, OrganoCat, OrganoSolv, Consolidated bioprocessing

## Abstract

**Background:**

Pretreated lignocellulosic biomass is considered as a suitable feedstock for the sustainable production of chemicals. However, the recalcitrant nature of cellulose often results in very cost-intensive overall production processes. A promising concept to reduce the costs is consolidated bioprocessing, which integrates in a single step cellulase production, cellulose hydrolysis, and fermentative conversion of produced sugars into a valuable product. This approach, however, requires assessing the digestibility of the applied celluloses and, thus, the released sugar amount during the fermentation. Since the released sugars are completely taken up by *Trichoderma reesei* Rut-C30 and the sugar consumption is stoichiometrically coupled to oxygen uptake, the respiration activity was measured to evaluate the digestibility of cellulose.

**Results:**

The method was successfully tested on commercial cellulosic substrates identifying a correlation between the respiration activity and the crystallinity of the substrate. Pulse experiments with cellulose and cellulases suggested that the respiration activity of *T. reesei* on cellulose can be divided into two distinct phases, one limited by enzyme activity and one by cellulose-binding-sites. The impact of known (cellobiose, sophorose, urea, tween 80, peptone) and new (miscanthus steepwater) compounds enhancing cellulase production was evaluated. Furthermore, the influence of two different pretreatment methods, the OrganoCat and OrganoSolv process, on the digestibility of beech wood saw dust was tested.

**Conclusions:**

The introduced method allows an online evaluation of cellulose digestibility in complex and non-complex cultivation media. As the measurements are performed under fermentation conditions, it is a valuable tool to test different types of cellulose for consolidated bioprocessing applications. Furthermore, the method can be applied to identify new compounds, which influence cellulase production.

**Electronic supplementary material:**

The online version of this article (doi:10.1186/s12934-016-0567-7) contains supplementary material, which is available to authorized users.

## Background

As the world’s most abundant and renewable natural resource lignocellulosic biomass is a potential feedstock to replace petroleum in a wide range of fossil-based products like fuels and chemicals [1]. Lignocellulose is the structural component of plant cell walls and mainly consists of cellulose (35–50 % (w/w)), which is associated with hemicellulose (20–35 % (w/w)) and interlinked by lignin (5–30 % (w/w)). Cellulose itself is a linear polymer built up of β-1,4 linked D-anhydroglucopyranose molecules with a degree of polymerization between 100 and 15,000 molecules. Due to the linear structure of the polymer adjacent cellulose chains align in a parallel fashion building up a highly ordered crystalline tertiary structure. The cellulose fibrils possess high tensile strength and low accessibility for degrading enzymes and chemicals making them difficult to convert. Some parts of the fibrils, however, are less ordered and referred to as amorphous regions [2]. As a result, these amorphous regions provide improved accessibility for an enzymatic breakdown. Due to its complexity and rigid structure an economic and efficient enzymatic conversion of the cellulose fraction to glucose still remains a bottleneck for the use of lignocellulosic biomass [3, 4].

Four processing steps are needed to convert lignocellulosic biomass to a valuable product: biomass pretreatment, enzymatic hydrolysis of the carbohydrate fraction, fermentation of sugars to products and downstream processing [5]. The pretreatment mostly includes a mechanical disintegration step such as hammer or ball milling followed by a chemical or thermochemical process. Examples of chemical steps include alkaline pretreatment with sodium or ammonium hydroxide, acid pretreatment using sulfuric acid, oxalic acid or peracetic acid, and a combination of organic solvents with acids as in the OrganoSolv or OrganoCat process [5–7]. The biomass pretreatment facilitates enzymatic hydrolysis by removing most of the lignin and in some cases hemicellulose, thus, increasing cellulose accessibility. Furthermore, the degree of polymerization as well as crystallinity of the cellulose is reduced. In all pretreatment processes by-products such as weak acids, furans, and phenolic compounds can be produced [8]. As a result, the type of pretreatment can influence to a great extent the subsequent hydrolysis and fermentation step.

Cellulose is hydrolyzed by a cocktail of different enzymes called cellulases. For the conversion three different enzymatic specificities are essential: endoglucanases (EC 3.2.1.4), cellobiohydrolases (EC 3.2.1.91), β-glucosidases (EC 3.2.1.21) [9]. The overall hydrolytic efficiency of the enzyme cocktail depends on the properties of the individual enzymes and their ratio in the cocktail. Furthermore, the substrate properties determine the rate of hydrolysis. For pure cellulosic substrates the particle size, degree of polymerization, crystallinity, accessible surface area, as well as the pore size are regarded as important characteristics [10]. In pretreated biomass the hydrolysis is additionally affected by the remaining structural components lignin (mainly) and hemicellulose. The exact interconnection of physicochemical properties with the rate of hydrolysis is still not completely understood [11, 12].

Cellulases are produced by bacteria like *Clostridium* and *Actinomycetes* species as well as filamentous fungi belonging to the genus of *Aspergillus*, *Penicillium* or *Trichoderma* [13]. The mesophilic soft-rot fungus *Trichoderma reesei* secretes the enzymes and is one of the major microorganisms for cellulase production in academic research and the industrial sector [14]. Enzyme production is strongly influenced by the kind of carbon source and culture conditions like aeration, temperature, and pH value [15–17]. Furthermore, cellulase synthesis is tightly regulated by induction and product (especially glucose) inhibition. Cellulase expression is induced by the natural substrate cellulose but also activated by some of its hydrolytic products and certain oligosaccharides. Examples of inducers are cellobiose, lactose, or sophorose [18].

Since enzyme production is a very expensive step in the conversion of cellulosic biomass, strategies are needed to decrease its costs [19]. Consolidated bioprocessing (CBP) offers great potential to substantially decrease costs by combining cellulase production, hydrolysis, and fermentation of sugars in one reaction step. This can be achieved by using engineered microorganisms capable of producing a suitable cellulase cocktail as well as the desired product. Another strategy is to use a microbial consortium dedicated to the different tasks. Although CBP is a highly promising approach, examples of successful integration of the different steps are scarce [20–25]. This is probably due to the high complexity of the resulting system, which is characterized by its enzyme production as well as cellulose degradation and product formation characteristics.

**Fig. 1:**
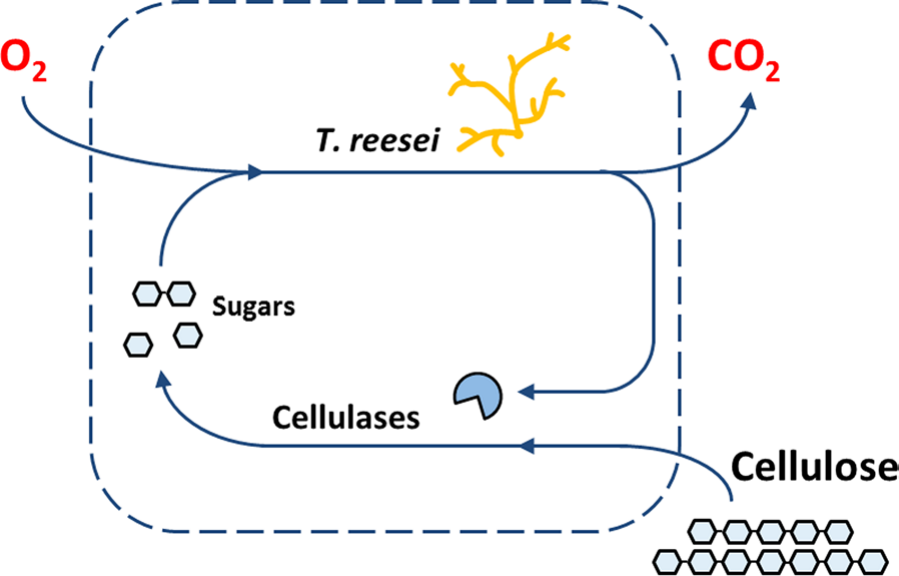
Process scheme of cellulose digestion by *T. reesei*. *T.* *reesei* consumes oxygen and the soluble sugars glucose and cellobiose, producing a mixture of cellulases and CO_2_. The solid substrate cellulose is hydrolyzed by the produced cellulases to yield soluble sugars, closing the loop. The *dashed line* symbolizes the boundary for material balances. Oxygen consumption is measured online. This concept can be used to investigate cellulose digestion because the cellulose hydrolysis is the limiting step and no soluble sugars accumulate during the whole reaction

As a first step to set up a consolidated bioprocess, the present study aimed at the development of a method to evaluate the digestibility of different cellulosic materials in situ. In this case, the measurements of cellulose digestion have to be performed using the chosen fermentation conditions, which determine the enzyme production as well as cellulose hydrolysis. Figure 1 shows a process scheme of cellulose digestion by *T.* *reesei*. The fungus consumes the sugars glucose and cellobiose to produce the cellulase cocktail. The sugars do not accumulate because cellulose hydrolysis is the limiting step during the whole reaction. Oxygen is taken up by the fungus and carbon dioxide is released, according to the stoichiometry of the reaction. The solid substrate cellulose is hydrolyzed by the produced cellulases to yield soluble sugars. Therefore, the respiration activity is a measure of the amount of the released sugars, which are produced by the secreted cellulase cocktail on the used type of cellulose under fermentation condition. After establishing a suitable cultivation procedure, four commercial cellulose materials were used as substrates to investigate their effect on the measured metabolic activity and, hence, their digestibility. The course of respiration activity during cellulose conversion was further analyzed by addition of cellulolytic enzymes and fresh cellulose substrate. Subsequently, the effect of inducers (cellobiose, sophorose), several media supplements (urea, tween 80 and peptone), and miscanthus steepwater, a possible by-product of a biorefinery, on the respiration activity was assessed. Finally, differently pretreated substrates, OrganoSolv and OrganoCat cellulose fractions from beech wood biomass fractionations, were tested for their suitability as cellulosic material for CBP.

## Results and discussion

### Effects of glucose and glycerol on the respiration activity of *T. reesei* Rut-C30

The production of cellulases is strongly influenced by the applied carbon source. If cellulose, a strong natural inducer of cellulase production, is used as sole carbon source, the initial spore germination will be strongly delayed. Therefore, complex compounds like peptone or yeast extract are often included in the culture broth to initiate growth [18, 26]. However, to avoid the influence of lot-to-lot variations of complex compounds during process characterization, their use should if possible be avoided [27]. Instead, two different carbon sources, namely glycerol and glucose, were tested to minimize the lag phase of the cultivation. Although glucose is a repressor for the production of cellulases, it is often included in pre-culture media [15]. In contrast, glycerol does not influence the production of cellulases [18, 28]. To monitor the growth in shake flasks in presence of a solid substrate, the respiration activity monitoring system (RAMOS) was used.

**Fig. 2:**
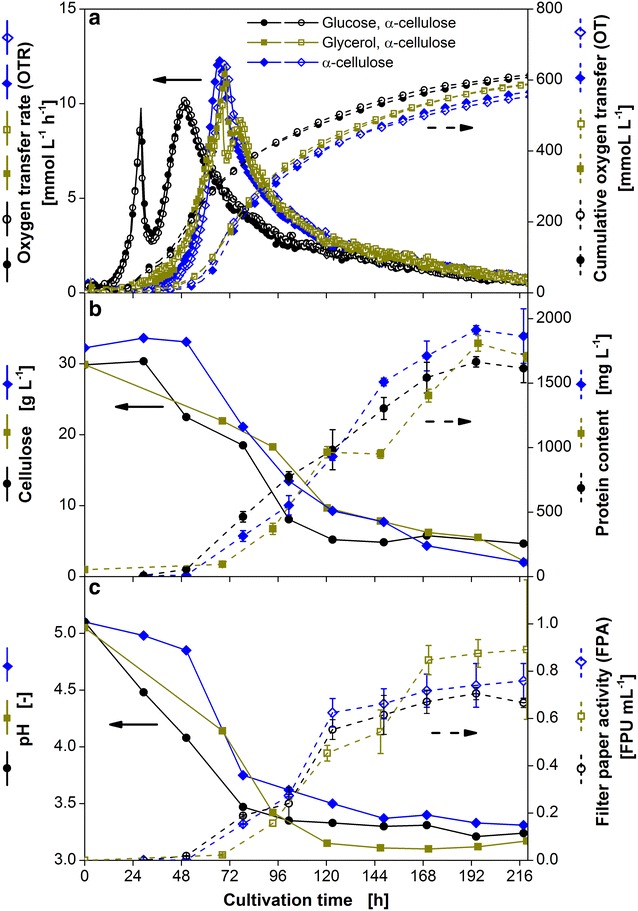
Effects of glucose and glycerol on the respiration activity of *T. reesei* Rut-C30. *T.* *reesei* Rut-C30 was grown on α-cellulose as sole carbon source (34.5 g L^−1^) and α-cellulose (30 g L^−1^) in combination with glucose (5.0 g L^−1^) or glycerol (5.1 g L^−1^). All cultures contained the same molar amount of carbon. **a** Duplicates of oxygen transfer rate (OTR) and cumulative oxygen transfer (OT). For clarity only every second measuring point over time is represented by a symbol; **b** cellulose and protein content in the culture supernatant; **c** pH and filter paper activity (FPA). *Error bars* represent standard deviation of technical triplicates. Culture conditions: modified Pakula medium, 250 mL flask, filling volume 20 mL, shaking frequency 350 rpm, shaking diameter 50 mm, inoculum 10^6^ spores mL^−1^, and 30 °C

Figure 2a shows the oxygen transfer rate (OTR) and cumulative oxygen transfer (OT) over time of a *T.* *reesei* Rut-C30 culture in a mineral medium with α-cellulose (34.5 g L^−1^) as sole carbon source and α-cellulose in combination with glucose (5.0 g L^−1^) or glycerol (5.1 g L^−1^). The amount of different carbon sources was adapted to yield the same overall amount of carbon. The measurements were performed in duplicates and the low variations prove the excellent repeatability of the cultivation in one parallel experiment. This is especially remarkable, as *T.* *reesei* is a filamentous fungus, which in general often show a quite variable morphology and behavior. However, it has already been shown that *T. reesei* can also be reproducibly cultured in microtiter plates [29]. The reproducibility of experiments performed at different time points is lower and is shown in the Additional file 1: Figure S1. Due to the detected intrinsic variability of the biological system, comparison of different cultivation conditions was always performed using only data from parallel experiments.

The culture containing α-cellulose as sole carbon source has the longest lag phase of about 45 h. Afterwards, the OTR increases to a maximum of 12 mmol L^−1^ h^−1^ after 70 h and then decreases very slowly over a period of 3–4 days in the further course of the cultivation. High cellulose consumption (Fig. 2b) and a steep drop in pH (Fig. 2c) coincide with the increase in OTR. In contrast, low cellulose consumption and a decelerated decline in pH can be observed during the subsequent OTR decrease after 70 h. In comparison, a culture supplemented with glycerol has a slightly shorter lag phase, but reaches the same maximum OTR at a similar time point. The maximum is followed by a short instantaneous drop and an increase in OTR reaching a second maximum of about 10 mmol L^−1^ h^−1^ after 80 h. This is a characteristic course of OTR for diauxic growth on two carbon sources [30]. The analysis of cumulative oxygen transfer (OT) and the course of cellulose consumption reveal that cellulose and glycerol are consumed simultaneously. The drop in OTR at about 70 h is probably caused by the exhaustion of glycerol as carbon source. The rate of final OTR decrease is similar to that of the culture with solely α-cellulose. The culture containing glucose has the shortest lag phase and reaches its first maximum of 10 mmol L^−1^ h^−1^ after 28, 42 h earlier compared to the culture with cellulose as sole carbon source. Furthermore, a similar two peak pattern as with glycerol is observed except for a steeper OTR decrease between the two maxima reaching a minimum of 3 mmol L^−1^ h^−1^. HPLC analysis confirms glucose is depleted at this local minimum (data not shown). Figure 2b illustrates that the consumption of cellulose in the culture with glucose, which is indicated by the second increase in OTR, does not set in until the initial glucose is consumed. Nonetheless, cellulose consumption starts about 10 h earlier compared to the culture supplemented with glycerol or solely with cellulose.

The OT of all three cultures reaches a final value of around 600 mmol L^−1^, indicating that the same amount of carbon was taken up. This is confirmed by the fact that the remaining cellulose concentration is around 4 g L^−1^ in all cultures. Additionally, the same final pH value of roughly 3.3 is reached.

The main proteins secreted by *T. reesei* Rut-C30 are cellulases. Therefore, the extracellular protein content correlates with the amount of cellulases produced [31]. As shown in Fig. 2b, a total protein concentration (Bradford) between 1600 and 1800 mg L^−1^ were reached in all cultures at the end of the fermentation. The final cellulase activity measured by the filter paper activity assay was around 0.7–0.9 filter paper units (FPU) mL^−1^ for all cultures, the culture with glycerol reaching slightly higher values (Fig. 2c).

The comparison of all three cultures reveals that the lag phase can be significantly shortened by addition of glucose, which is in good agreement with Zhang et al. [32]. The addition of glycerol influences the lag phase only slightly. It has to be noted that cellulose and glycerol were consumed simultaneously in contrast to glucose and cellulose, which were taken up consecutively. The reason for this might be the repressing effect of glucose on cellulase production [18]. No significant influence of glucose on the quantity of enzymes produced by *T. reesei* Rut-C30 was detected, however, enzyme production started at an earlier time point. This might be due to the fact that the addition of glucose results in a higher biomass formation allowing a faster cellulase production after the depletion of glucose. This assumption is supported by the earlier second rise in OTR starting at about 35 h caused by cellulose consumption. Szijarto et al. [33] performed pulse experiments, adding cellulose to cultures grown on glucose. He suggested to perform two stage cultivations, first on glucose for biomass formation, and afterwards using cellulose for enzyme production. The results obtained in this study suggest that via glucose addition to a culture containing cellulose, biomass growth and enzyme production can be realized in a single stage. Therefore, further experiments were performed using both glucose and cellulose as carbon sources to achieve a faster initiation of cellulose consumption.

### Digestibility of different commercial celluloses

The physical properties of the utilized celluloses influence induction of cellulase production as well as the rate of hydrolysis, thus determining their biological digestion rate [11, 15].

**Fig. 3:**
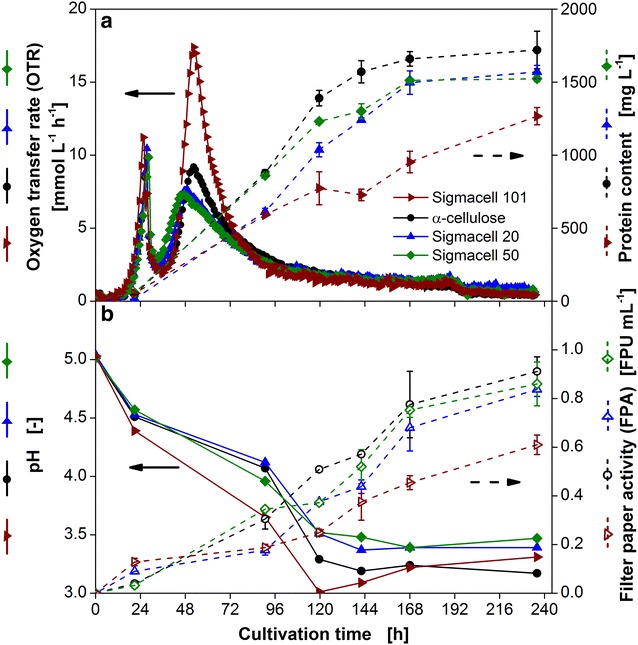
Characteristic growth and enzyme production parameters of *T.* *reesei* Rut-C30 on different types of cellulose. *T. reesei* Rut-C30 was grown on 5 g L^−1^ glucose and 30 g L^−1^ Sigmacell 101 (amorphous)/α-cellulose (Crl 41.5 %)/Sigmacell 20 (Crl 52.6 %)/Sigmacell 50 (Crl 56.1 %). **a** Oxygen transfer rate (OTR) and protein content in the culture supernatant. The OTR was measured in duplicates with a maximum mean error of 0.4 mmol L^−1^ h^−1^. For clarity of the depicted average values, only every second measuring point of OTR over time is represented by a symbol; **b** pH and filter paper activity. *Error bars* represent standard deviation of technical triplicates. Culture conditions: modified Pakula medium, 250 mL flask, filling volume 20 mL, shaking frequency 350 rpm, shaking diameter 50 mm, inoculum 10^6^ spores mL^−1^, and 30 °C

To evaluate the influence of cellulose with different physicochemical properties on the respiration activity of *T. reesei* Rut-C30, four commercially available celluloses, namely α-cellulose, Sigmacell 101, Sigmacell 20, and Sigmacell 50, were tested. Their crystallinity as well as particle size are given in Table. 1. All cultures contained 5 g L^−1^ glucose and 30 g L^−1^ cellulose. Figure 3a depicts the course of OTR over time. As expected, the observed pattern for the culture containing glucose and α-cellulose is very similar compared to the results shown in Fig. 2a. Regardless of the type of cellulose the OTR profiles exhibit a two peak pattern. The first maximum of 10 mmol L^−1^ h^−1^, corresponding to glucose consumption, is reached after 24 h of cultivation for all cultures. The second peak has a different shape depending on the type of cellulose investigated. The second increase in OTR for the culture on α-cellulose (Crl 41.5 %) and Sigmacell 101 (amorphous) starts simultaneously. However, the maximum OTR reached on Sigmacell 101 is twice as high as for the culture on α-cellulose. The OTR maximum further decreases for Sigmacell 20 (Crl 52.6 %) and Sigmacell 50 (Crl 56.1 %) to about 8 and 7 mmol L^−1^ h^−1^, respectively. All in all, at a higher crystallinity of cellulose the height of the second peak decreases. This observation indicates that the shape of the OTR curve can be used to describe the digestibility characteristics of the applied celluloses. For the tested celluloses no correlation between the particle size and the shape of the OTR curve was observed.

**Table 1:** Physical properties of the used types of cellulose and oxygen transfer-based parameters

	CrI [%]	d_p_ [µm]	Slope of linear increase [mmol L^−1^ h^−2^]	Slope of linear decrease [mmol L^−1^ h^−2^]	Area ratio X/Y [−]
Sigmacell 101	Amorphous^b^	15.68^b^	1.25	−0.70	0.59
α-Cellulose	41.5^a^	68.77^b^	0.58	−0.20	0.43
Sigmacell 20	52.6^a^	20^c^	0.44	−0.14	0.41
Sigmacell 50	56.1^a^	50^c^	0.39	−0.12	0.36

**Fig. 4:**
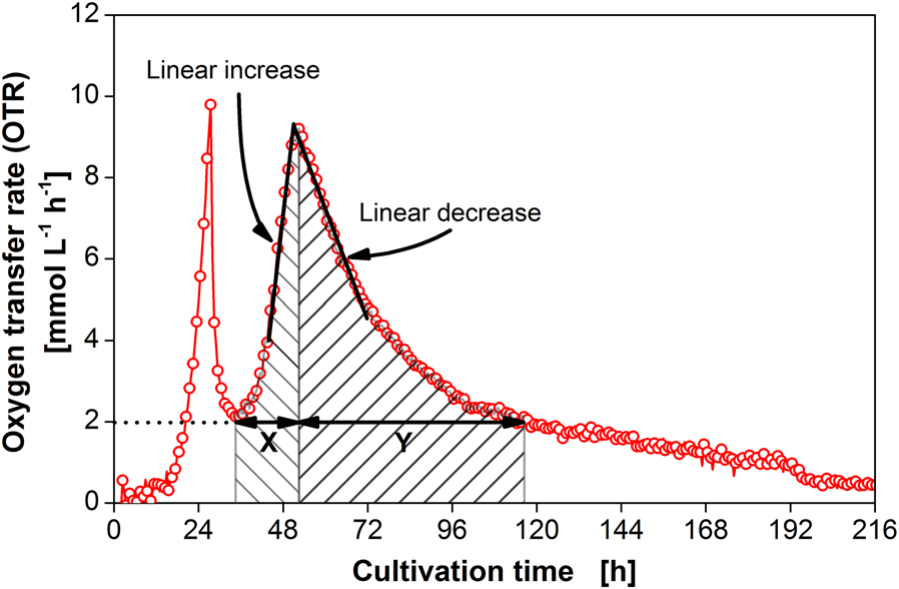
Illustration of areas of increasing (X) and decreasing (Y) oxygen transfer rate during cellulose utilization. *T.* *reesei* Rut-C30 was grown on 5 g L^−1^ glucose and 30 g L^−1^ α-cellulose as carbon sources. As lower threshold value for the calculation of the area of decreasing OTR an OTR = 2 mmol L^−1^ h^−1^ was chosen. The slopes of linear increase and linear decrease are marked by *black lines*

The crystallinity index (CrI) and particle size (d_p_) of the used types of cellulose are listed together with the slope of linear increase, slope of linear decrease as well as the area ratio of increasing (X) to the decreasing (Y) OTR according to Fig. 4. The values were calculated for a *T.* *reesei* Rut-C30 cultivation on 5 g L^−1^ glucose and 30 g L^−1^ of the corresponding type of cellulose as carbon sources

^a^NMR based calculation [62]

^b^Ref. [54]

^c^According to manufacturer’s data

To describe the different shapes of the second OTR peak, three different parameters are suggested according to Fig. 4: the slope of linear increase, slope of linear decrease, as well as the area ratio of increasing (X) to the decreasing (Y) OTR. The results are presented in Table. 1. The absolute value of the slope of linear increase and decrease, declines with increasing crystallinity of cellulose. It can be assumed that a faster change in degradation rate of cellulose leads to a higher amount of released sugar, resulting in a steeper OTR slope. As shown in the Additional file 2: Figure S2 the calculated slopes are strongly interlinked with each other and with the area ratio (X/Y). Therefore, only one of the parameters is needed to describe the characteristics of the peak.

Weimer and Weston [34] measured the rate of hydrolysis of the same types of celluloses applied in this work. The authors observed the same order in enzymatic digestibility as is shown using the OTR slopes, except for α-cellulose. The type of α-cellulose was, however, not specified, which impedes a comparison of results. Furthermore, it has to be noted that the cellulose digestion rate in the fermentation is not only influenced by the rate of enzymatic hydrolysis for a given amount of enzyme, but also by the amount of enzymes produced and their composition. As different types of cellulose influence the induction of enzyme production, it is not possible to determine the digestibility of cellulose by an organism simply by performing enzymatic tests [35]. Additionally, the usually conducted filter paper assay to determine the overall cellulase activity is conducted under optimal conditions for the enzymatic reaction and not under fermentation conditions.

The effect of different enzyme production profiles can be clearly seen in Fig. 3. The protein content (Fig. 3a) and the filter paper activity (Fig. 3b) of all crystalline types of cellulose are quite similar. The highest protein content and filter paper activity is reached using α-cellulose with around 1700 mg L^−1^ and 0.9 FPU mL^−1^, respectively. The culture with Sigmacell 101, the amorphous cellulose, only reaches a protein content of about 1200 mg L^−1^ and a filter paper activity of 0.6 FPU mL^−1^. This is probably due to the fact that amorphous cellulose is easier to degrade, causing an enhanced glucose supply and, thereby, leading to repression of cellulase synthesis [36]. Additionally, the pH value is also an important parameter for cellulase production. The slightly lower pH values of the culture with Sigmacell 101 could also lead to lower cellulase production.

Besides the differences in the OTR slopes also the area ratio of increasing (X) to the decreasing (Y) OTR during cellulose utilization was analyzed (Table 1). The area ratio decreased with increasing crystallinity. In order to understand this correlation, reasons for the increase and decrease in OTR are analyzed in the following section.

### Identification of crucial factors determining cellulose digestibility

In a standard fermentation with readily available soluble carbon sources a steep drop in OTR generally coincides with the exhaustion of a carbon source [30]. However, during the second growth phase on cellulose the OTR decreases although cellulose is still present. Furthermore, the decline of respiration activity during the growth on cellulose is much slower (3–4 days) compared to the decline correlated with the exhaustion of excessive glucose (2–3 h). To investigate the reasons for the observed respiration behavior, pulse experiments with cellulolytic enzymes and cellulose were performed.

**Fig. 5:**
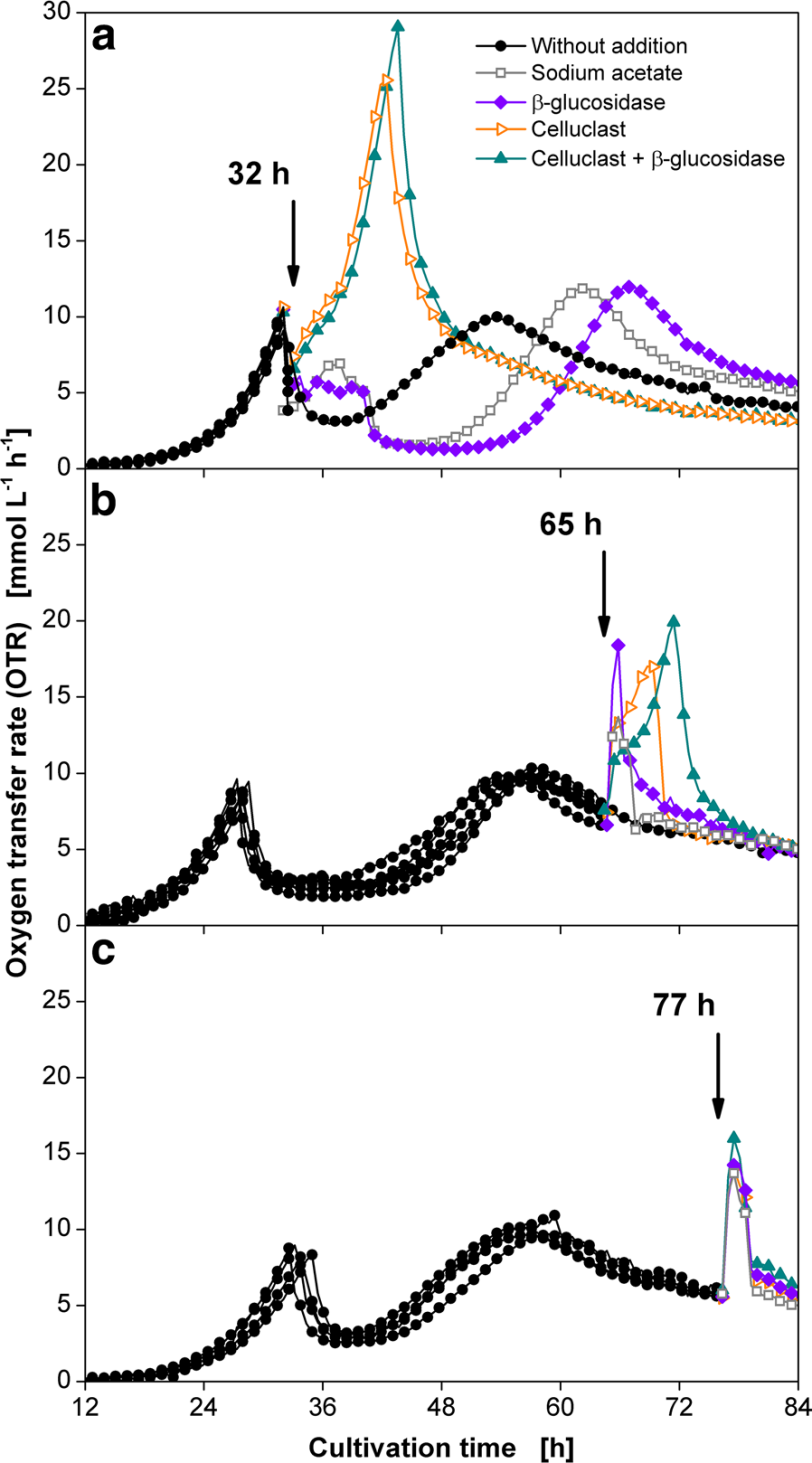
Addition of cellulases at different time points to the culture of *T.* *reesei* Rut-C30. *T.* *reesei* Rut-C30 was grown on 5 g L^−1^ glucose and 30 g L^−1^ α-cellulose. Oxygen transfer rate (OTR) after addition of 6 IU mL^−1^ of β-glucosidase, 6 FPU mL^−1^ Celluclast or the combination of both enzymes at 32 h (**a**), 65 h (**b**) and 77 h (**c**). For comparison, cultures without any addition and cultures with addition of 1 mL 0.1 M sodium acetate buffer (pH 4.8), used as solvent for the enzymes, are shown. The start of the time axes was set to 12 h to increase the visibility of the results. Prior to enzyme addition, all curves are depicted as *black circles* like the reference cultivation. Addition time points are marked by *arrows*. For clarity only every second measuring point over time is represented by a symbol. Culture conditions: modified Pakula medium, 250 mL flask, filling volume 20 mL, shaking frequency 350 rpm, shaking diameter 50 mm, inoculum 10^6^ spores mL^−1^, and 30 °C

Figure 5a depicts the addition of β-glucosidase, Celluclast 1.5 L or the combination of both enzyme solutions at 32 h, shortly after the drop in OTR caused by the exhaustion of glucose. As a reference, cultivations without any additives and with addition of 1 mL 0.1 M sodium acetate buffer (pH 4.8), which is contained in the enzyme solutions, are shown. The addition of sodium acetate leads to an increase in OTR to about 7 mmol L^−1^ h^−1^ until a sudden drop in OTR at 41 h. Furthermore, the increase in OTR corresponding to the initiation of cellulose conversion is retarded reaching the second peak 9 h later than for the reference cultivation. No explanation of this behavior could yet be found. The addition of β-glucosidases has no effect on cellulose digestion as the same OTR pattern is observed as for the addition of sodium acetate buffer. The injection of Celluclast or Celluclast supplemented with β-glucosidases leads to a fast rise in OTR reaching 25 mmol L^−1^ h^−1^ and 30 mmol L^−1^ h^−1^, respectively. This indicates that the time point of increase in OTR after glucose consumption is determined by the availability of cellulases.

The same procedure was performed after the second OTR peak at 65 h (Fig. 5b) of cultivation. The addition of sodium acetate leads to a peak in OTR corresponding to the oxidative consumption of the substance. The addition of Celluclast and Celluclast with β-glucosidases results in a fast initial OTR increase like in the case of sodium acetate addition. It is followed by a slower increase in OTR reaching a maximum of 18 and 21 mmol L^−1^ h^−1^, respectively. This difference in OTR indicates a slight limitation of the β-glucosidase in the enzyme cocktail produced by *T. reesei*. It is known that *T. reesei* Rut-C30 is deficient in β-glucosidases [37]. The increase in OTR can be attributed to an increased hydrolysis rate by the added enzymes. Thereby, not only the higher amount of enzymes might be the reason for the increased hydrolysis rate, but also the different composition of the commercial enzyme cocktail could allow a more efficient conversion of the remaining cellulose. Afterwards, the OTR drops to the same OTR value like the culture without any supplements. When the enzymes are added even later during the OTR decrease (at 77 h), no effect of enzyme addition can be seen at all (see Fig. 5c), as the respiration activity is equivalent to that of the acetate solution. At this time point, the amount of available enzymes is not the crucial factor limiting the respiration activity of the culture.

The decline in OTR during the fermentation resemble the decrease in enzymatic cellulose hydrolysis at high degrees of conversion. Several factors were suggested, which could play an important role in the detected decline of the reaction rate: product inhibition, inactivation of cellulases, alteration of cellulose accessibility or reactivity [11, 38, 39]. The physicochemical reason for the decrease in cellulose accessibility or reactivity is still a subject of scientific discussions. As no glucose or cellobiose was detected in the supernatant of the cultures, the first reason was ruled out in the present case. In order to check the influence of the other factors, fresh cellulose was added as a substrate at different time points.

**Fig. 6:**
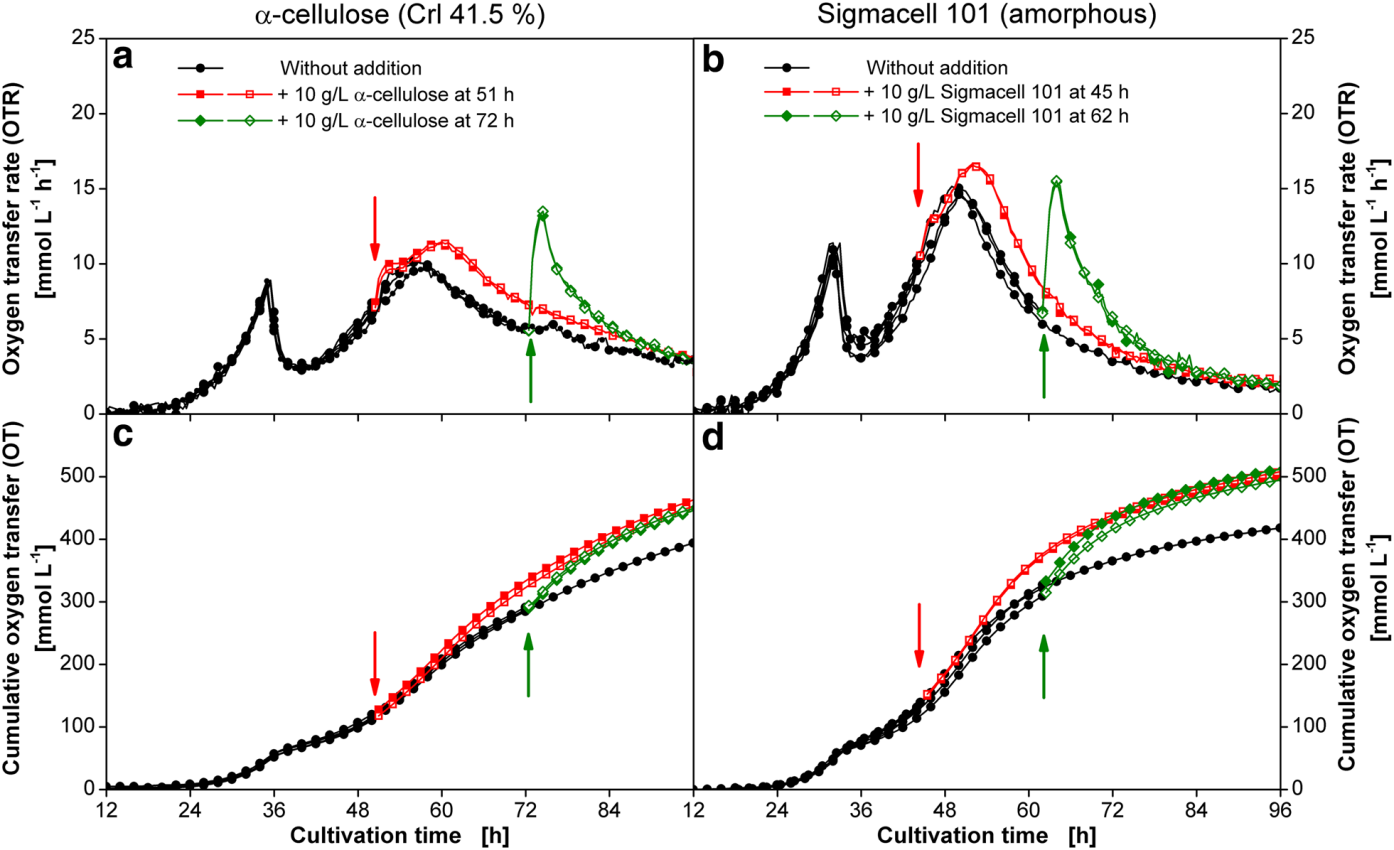
Addition of fresh cellulose at different time points to the culture of *T.* *reesei* Rut-C30. *T.* *reesei* Rut-C30 was grown on 5 g L^−1^ glucose and 30 g L^−1^ cellulose. **a** Duplicates of oxygen transfer rate (OTR) and **c** cumulative oxygen transfer (OT) during growth on α-cellulose. 10 g L^−1^ α-cellulose was added after 51 and 72 h of cultivation to different shake flasks; **b** duplicates of oxygen transfer rate (OTR) and **d** cumulative oxygen transfer (OT) during growth on Sigmacell 101. 10 g L^−1^ Sigmacell 101 was added after 45 and 62 h of cultivation to different shake flasks. The start of the time axes was set to 12 h to increase the visibility of the results. Prior to cellulose addition, all curves are depicted as *black circles* like the reference cultivation. Addition time points are marked by *arrows*. For clarity only every fourth measuring point over time is represented by a symbol. Culture conditions: modified Pakula medium, 250 mL flask, filling volume 20 mL, shaking frequency 350 rpm, shaking diameter 50 mm, inoculum 10^6^ spores mL^−1^, and 30 °C

In Fig. 6 the OTR of pulse experiments performed during growth on crystalline α-cellulose (a) and amorphous Sigmacell 101 (b) are shown. All experiments performed in duplicates confirm the excellent repeatability of the results. The corresponding type of cellulose was added during the increase or decrease in OTR of the second OTR peak, respectively. The addition of 10 g L^−1^ cellulose after 51 h for α-cellulose and 45 h for Sigmacell 101 has nearly no immediate influence on the OTR curve. However, the increase in OTR ceases later and a higher second OTR maximum is reached, as more cellulose is present in the medium. Thus, it can be concluded, that at this stage before the OTR maximum enough well digestible substrate is present and the conversion is limited by the amount of cellulases available (see Fig. 5). In contrast, the addition of cellulose after the second OTR maximum causes an immediate strong increase in OTR from 5 mmol L^−1^ h^−1^ to 14 (Fig. 6a) or 15 mmol L^−1^ h^−1^ (Fig. 6b), respectively. After the fast strong increase, a slow decrease follows reaching a similar OTR value like the culture without any additions. In Fig. 6c and d the corresponding cumulative oxygen transfers (OT) of the cultures are shown. Irrespective of the time point of addition of cellulose, the same OTs were measured after 96 h of fermentation for the same type and amount of cellulose used. In total, the digestion of the amorphous cellulose leads to a slightly higher OT after 96 h of cultivation (500 vs 440 mmol L^−1^) indicating a higher degree of conversion.

The immediate reaction upon cellulose addition after the second OTR peak rules out the inactivation of cellulases to be the reason for the decrease in OTR when cellulose is still present (Fig. 6). Furthermore, the results reveal that besides the available amount of carbon source, no other nutrients in the culture medium are limiting. The reason for the OTR drop might be a change in cellulose accessibility due to a limitation of free binding sites, which might lead to the so called jamming effect among cellulases [40, 41]. This hypothesis is supported by the higher absolute level of OTR decrease of the cultures supplemented with additional cellulose during their OTR increase compared to the cultures without any additions. Therefore, the phase of increasing OTR is characterized by easily digestible cellulose utilization, so that the enzyme amount limits cellulose conversion. The subsequent phase of decreasing OTR is most probably caused by a decreased substrate accessibility. As substrate accessibility is influenced by the crystallinity of cellulose, this could be the reason for the correlation observed between the area ratio of increasing (X) to the decreasing (Y) OTR and the crystallinity of the substrate (see Table 1). The area ratio might provide information about the easily digestible and hardly accessible cellulose part.

### Influence of complex and non-complex compounds on cellulase production

Cellulase production is influenced by a variety of media compounds. In the first instance, cellulase inducers were added to the fermentation broth to evaluate the effect of enhanced cellulase production on the respiration activity. As inducer compounds, a weak inducer (cellobiose) and a strong inducer (sophorose) were chosen.

**Fig. 7:**
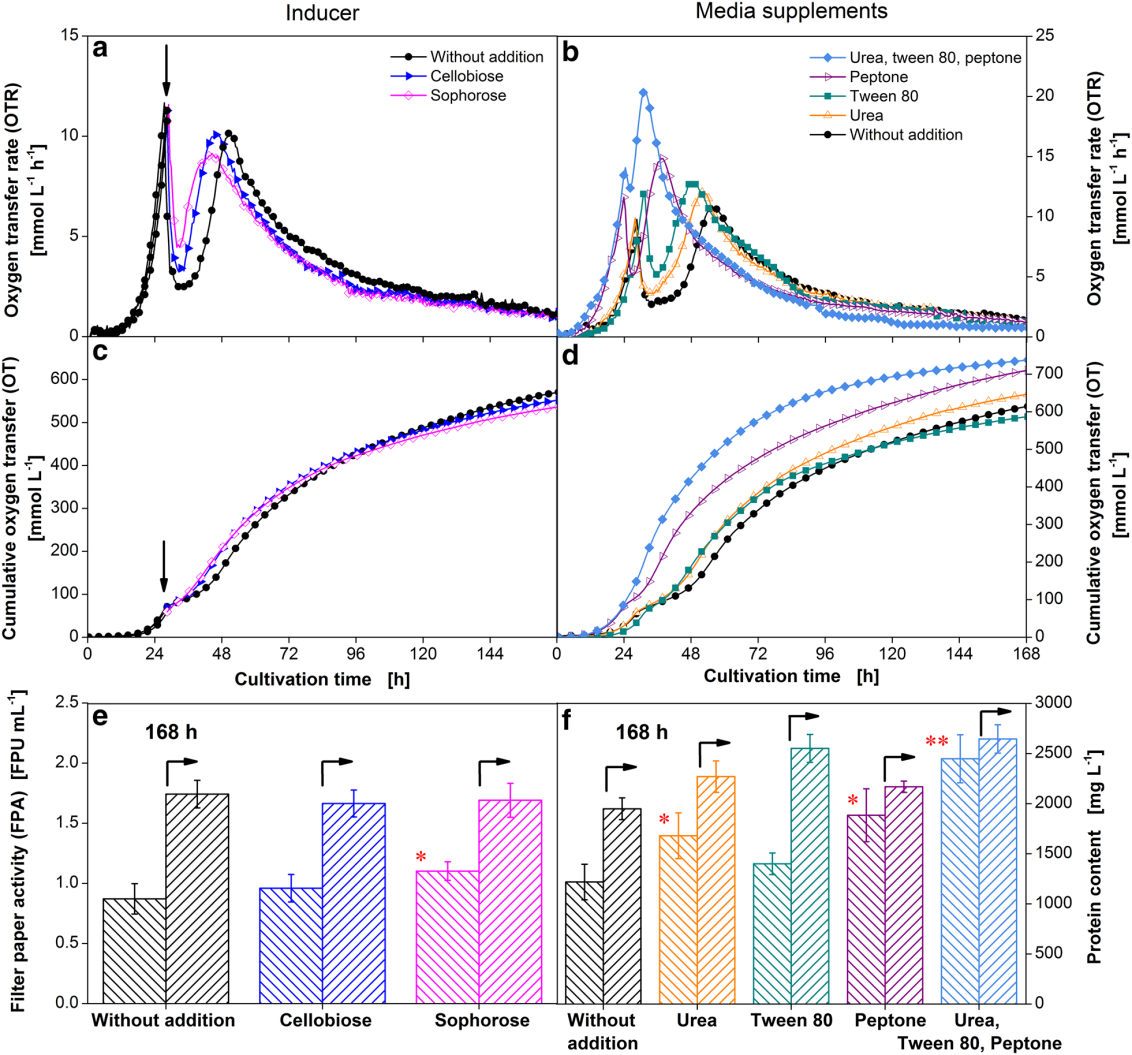
Addition of different cellulase inducers and media supplements to the culture of *T.* *reesei* Rut-C30. *T. reesei* Rut-C30 was grown on 5 g L^−1^ glucose and 30 g L^−1^ α-cellulose. Oxygen transfer rate (OTR), cumulative oxygen transfer (OT), filter paper activity and protein content in the culture supernatant of a culture without and with addition of 0.24 g L^−1^ (0.7 mm) cellobiose or sophorose after 28 h of cultivation (**a, c, e**) and a culture medium supplemented with 0.3 g L^−1^ urea, 0.1 % (v/v) tween 80, 2 g L^−1^ peptone or the mix of all three compounds (**b**, **d**, **f**). The filter paper activity and the protein content in the culture supernatant were measured after 168 h. The OTR was measured in duplicates. As the maximum mean error was as low as 0.5 mmol L^−1^ h^−1^, average values are shown. For clarity only every fourth (OTR) and every eighth (OT) measuring point over time is represented by a symbol in (**a**–**d**), respectively. In **e** and **f** an *asterisk* marks a significant increase in filter paper activity compared to a culture without additions with significance level of 95 %, *two asterisks* correspond to a level of 99 %. *Error bars* represent standard deviation of technical triplicates. Culture conditions: modified Pakula medium, 250 mL flask, filling volume 20 mL, shaking frequency 350 rpm, shaking diameter 50 mm, inoculum 10^6^ spores mL^−1^, and 30 °C

Figure 7a illustrates the respiratory activity of a culture without any supplements and with addition of cellobiose or sophorose. In comparison to the reference cultivation, the addition of cellobiose causes a reduction in time of 4.5 h between the peaks corresponding to glucose consumption and the peak of cellulose digestion. The same amount of sophorose (0.7 mM) has an even stronger influence reducing the time by 7 h. The faster metabolic shift can also be observed considering the elevated OTR minimum between the two peaks. At the same time, the further course of the OTR over time is not influenced. The addition of inducers does not significantly change the overall amount of oxygen consumed, as shown in Fig. 7c.

In Fig. 7e the filter paper activity and the protein content of the corresponding approaches are shown after 168 h. The protein content is quite similar for all cultures. The measured filter paper activity is significantly increased for the culture containing sophorose.

Cellobiose has been considered to be a poor inducer, especially when it is available at high levels causing catabolite repression [28, 42]. Although the final amount of enzymes produced is not influenced, the effect of an earlier cellulase production can be clearly seen based on the OTR profile. Sophorose is the most potent cellulase inducer [43]. Thereby, an effect on the final filter paper activity and the OTR curve is explained.

Besides substances, which directly influence the regulation of cellulase production on transcriptome level, the addition of some media supplements can lead to an increase in cellulase production. The influence of urea, tween 80, peptone and the combination of all three compounds on the OTR as a function of time is depicted in Fig. 7b. The addition of urea and tween 80 causes a negative shift of the second peak by 3 and 5 h, respectively, similar to the cellulose inducers cellobiose or sophorose. The final OT is quite similar among the culture without any additions and the cultures supplemented with urea and tween 80 (Fig. 7d). The measured protein content rises from 1950 mg L^−1^ for the reference culture to 2270 mg L^−1^ for the culture containing urea and 2520 mg L^−1^ for the culture supplemented with tween 80 (Fig. 7f). However, the measured filter paper activity in the culture supernatant after 168 h of cultivation does not follow this trend. The filter paper activity of the culture with tween 80 is lower (1.2 FPU mL^−1^) compared to the culture with urea (1.4 FPU mL^−1^). Furthermore, tween 80 is not significantly increasing the filter paper activity compared to the control. Tangnu et al. [37] also observed the positive effect of urea on enzyme production. The higher non-cellulolytic protein content in the supernatant of the culture supplemented with tween 80 was expected as tween 80 is supposed to increase the permeability of the cell membrane [37].

The supplementation of the culture medium with peptone leads to a 10 h shorter lag phase and a faster transition phase from glucose consumption to cellulose digestion. Furthermore, the second OTR maximum with a value of 15 mmol L^−1^ h^−1^ is 5 mmol L^−1^ h^−1^ higher compared to the culture without any additions. Thereby, a protein content of 2170 mg L^−1^ is reached corresponding to a filter paper activity of 1.6 FPU mL^−1^. The culture containing all three compounds shows a similar lag phase like the culture with peptone, but the metabolic shift from glucose consumption to cellulose digestion is much quicker. Only a slight drop in OTR is visible after the depletion of glucose. An even higher second OTR maximum of 20 mmol L^−1^ h^−1^ is reached. The comparison of the OT reveals a higher overall oxygen consumption for the cultures containing peptone compared to the reference cultivation. However, the difference can be attributed to the oxidative conversion of peptone. The positive effect of peptone on cellulase production is widely excepted and was analysed by Ilmén et al. [18].

As a cheap natural supplement, the influence of miscanthus steepwater on the cultivation of *T.* *reesei* Rut-C30 was tested. Miscanthus is a fast-growing perennial sweet grass also growing on marginal land. It is, therefore, suitable as a feedstock for a biorefinery. However, concerns were raised for the use of miscanthus, as the liquid waste after washing of miscanthus showed an inhibitory effect on the growth of seedlings (Winzer, University of Bonn, Germany, personal communication). Furthermore, an antimicrobial effect on *Pseudomonas* strains and *Clostridium beijerinckii* was detected [44, 45].

**Fig. 8:**
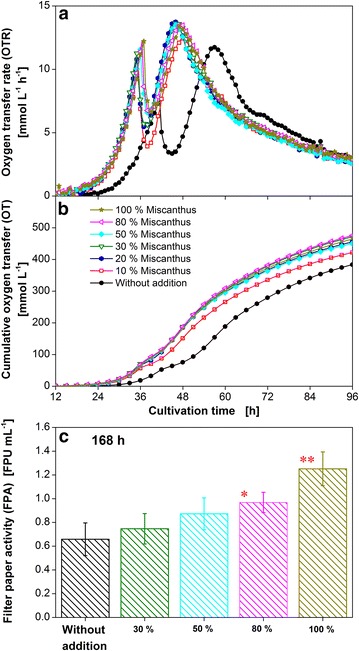
Characteristic growth and enzyme production parameters of *T.* *reesei* Rut-C30 using miscanthus steepwater. *T. reesei* Rut-C30 was grown on 5 g L^−1^ glucose and 30 g L^−1^ α-cellulose containing 10, 20, 30, 50, 80, 100 % (v/v) of miscanthus steepwater. **a** Oxygen transfer rate (OTR); **b** cumulative oxygen transfer (OT). For clarity only every second measuring point over time is represented by a symbol; **c** filter paper activity. A *asterisk marks* a significant increase in filter paper activity compared to a culture without additions with significance level of 95 %, two *asterisks* correspond to a level of 99 %. *Error bars* represent standard deviation of technical triplicates. The start of the time axes was set to 12 h to increase the visibility of the results. Culture conditions: modified Pakula medium, 250 mL flask, filling volume 20 mL, shaking frequency 350 rpm, shaking diameter 50 mm, inoculum 10^6^ spores mL^−1^, and 30 °C

The water for the preparation of the culture medium was replaced by miscanthus steepwater to different degrees (10–100 % (v/v)), to test its influence on *T. reesei* growth and cellulase production. According to Fig. 8a the addition of miscanthus steepwater for all tested concentrations resulted in a decrease of the lag phase by 5 h. Thus, the steepwater contains substances which have a positive effect on the germination of *T. reesei* spores. Furthermore, the time span between the two OTR peaks was reduced by 5 h, indicating an earlier start in enzyme production. The minimum between the two OTR peaks depends on the concentration of the miscanthus steepwater. From 10 to 50 % (v/v) of the miscanthus steepwater the OTR minimum increased continuously. Above 50 % only small differences between the different approaches were detected. The comparison of the OT (Fig. 8b) reveals that the addition of miscanthus steepwater increases the overall oxygen consumption from around 440–474 mmol L^−1^ for the culture containing 10 % (v/v) and approx. 520 mmol L^−1^ for all other concentrations of the miscanthus steepwater. In order to understand the influence of the miscanthus steepwater on the oxygen consumption of *T.* *reesei* Rut-C30, the liquid was analyzed by high pressure ion-exchange chromatography as well as liquid chromatography-mass spectrometry. The spectra were very complex and only few compounds could be identified. The glucose concentration was 0.3 and 0.03 mg L^−1^ xylose was detected. As these amounts are too low to explain the increase in OT, the total organic carbon was measured to reveal the overall potential of oxidizable substances. The total carbon content was 49 mg L^−1^. This value corresponds well with previously published data for a variety of natural surface waters [46]. However, the theoretical oxygen demand to oxidize this amount of carbon is around 9 mmol L^−1^ and, therefore, too low to explain the increase in OT of 80 mmol L^−1^. This suggests that more cellulose is consumed within 120 h when the fermentation medium is prepared with miscanthus steepwater. As shown in the Additional file 3: Figure S3, the presence of fluorescent substances like those found in natural surface waters, belonging to the class of poly-hydroxy-methoxy-carboxylic acids and quinones, was detected by 2D fluorescence spectra [46]. It can be speculated that these substances may have triggered a higher cellulase production and cellulose digestion. However, this explanation definitely requires further proof. In Fig. 8c the corresponding filter paper activity is shown. The filter paper activity increases continuously reaching 1.3 FPU mL^−1^ in a culture where the water in the medium is fully replaced by the miscanthus steepwater. Although the OTR changes between 50 and 80 % (v/v) of miscanthus steepwater are small, a big influence on the cellulolytic activity was detected. Surprisingly, despite the observed inhibitory effect on the growth of seedlings and *Pseudomonas* strains the use of miscanthus steepwater showed a positive effect on growth and cellulase production of *T. reesei*.

### Evaluation of digestibility of pretreated cellulosic substrates

For the economic production of cellulases at industrial scale it is not possible to use purified cellulose [47]. Therefore, the production of cellulases on pretreated plant waste material and pretreated lignocellulosic material is necessary. This aspect was addressed by several studies [36, 47, 48]. In contrast to these approaches, the aim of the current study was to monitor the digestion of cellulose and, hence, the amount of released sugars to estimate the potential of the substrates for consolidated bioprocessing. Thus, the digestibility of beech wood saw dust, pretreated by the OrganoCat and OrganoSolv process, respectively, was tested and compared to a culture containing α-cellulose. The media supplements (urea, tween 80 and peptone) showing a positive effect on cellulase production were added to the fermentation media.

**Fig. 9:**
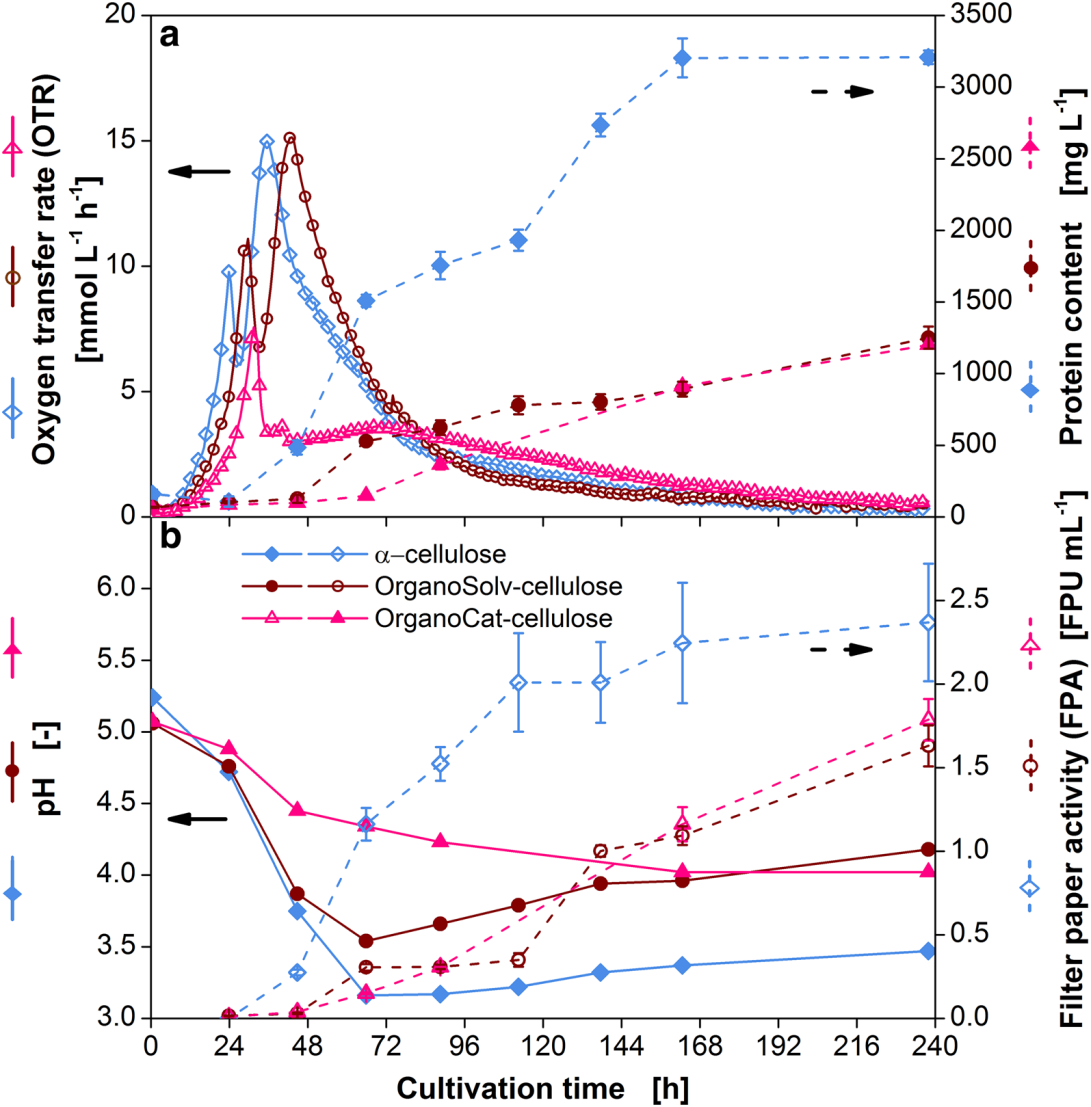
Characteristic growth and enzyme production parameters of *T.* *reesei* Rut-C30 on OrganoCat and OrganoSolv cellulose. *T. reesei* Rut-C30 was grown on 5 g L^−1^ glucose and 30 g L^−1^ α-cellulose, 33 g L^−1^ OrganoCat-cellulose, 33 g L^−1^ OrganoSolv-cellulose, supplemented with 0.3 g L^−1^ urea, 0.1 % (v/v) tween 80 and 2 g L^−1^ peptone. **a** Oxygen transfer rate (OTR) and protein content in the culture supernatant; **b** pH and filter paper activity. The OTR was measured in duplicates. As the maximum mean error was as low as 1 mmol L^−1^ h^−1^, average values are shown. For clarity only every second measuring point over time is represented by a symbol. *Error bars* represent standard deviation of technical triplicates. Culture conditions: modified Pakula medium, 250 mL flask, filling volume 20 mL, shaking frequency 350 rpm, shaking diameter 50 mm, inoculum 10^6^ spores mL^−1^, and 30 °C

As shown in Fig. 9a, the replacement of α-cellulose by the pretreated lignocelluloses cause a slight delay of the lag phase by 4 and 6 h for OrganoSolv and OrganoCat cellulose, respectively. This effect was previously observed on pretreated biomass and can be attributed to the production of inhibitory compounds during the pretreatment process [49]. The second peak, corresponding to the phase of cellulose digestion with OrganoSolv cellulose, is very similar compared to the peak for α-cellulose. Therefore, the amount of released sugars should agree quite well in both cases. A different behavior is observed for the culture containing OrganoCat cellulose. After the decrease in OTR caused by glucose exhaustion, the OTR drops rapidly to 3 mmol L^−1^ h^−1^ and increases only slightly to about 4 mmol L^−1^ h^−1^ in the further course of the fermentation. No distinct second peak is observed. Therefore, the conversion of OrganoCat cellulose is rather inefficient. This was confirmed by measuring the remaining cellulose content at the end of the fermentation. The culture with α-cellulose contained a residual amount of 1.7 g L^−1^ cellulose, the OrganoSolv approach contained 2.1 g L^−1^ cellulose, and the cellulose content in the OrganoCat culture was 11.2 g L^−1^ (data not shown). In Fig. 9b the pH profile of the three cultures is shown. The course of the pH exhibit higher values for the culture with OrganoSolv cellulose compared to the culture with α-cellulose. The reason for this difference is not clear as a similar cellulose consumption was observed. The pH of the culture containing OrganoCat cellulose decreases slowly and reaches a value of 4.0 similar to the pH value of the culture with OrganoSolv cellulose.

In addition to online analysis, the protein content and the filter paper activity were determined. The protein content as well as the filter paper activity of the culture with α-cellulose increased throughout the fermentation reaching a protein content of 3200 mg L^−1^ and a filter paper activity of 2.4 FPU mL^−1^ after 240 h, respectively. Thereby, most of the protein was produced during the OTR decrease. The decreased substrate accessibility causes carbon source limiting conditions enhancing cellulase production [50]. Although the OTR profile of the culture with OrganoSolv cellulose is quite similar, the measured protein content and filter paper activity are very different. Although the amount of proteins produced is 2.8 times lower, the measured filter paper activity is just 1.5 times lower. Therefore, the protein specific enzyme activity is higher for OrganoSolv and OrganoCat cellulose comparted to α-cellulose. This agrees quit well with findings by Doppelbauer et al. [51], who have shown that pretreated lignocellulosic substrates can cause a decrease in the observed filter paper activity. The enzyme production on OrganoCat cellulose sets in later, compared to the culture on OrganoSolv cellulose. Nevertheless, at the end of fermentation an equal amount of protein and the same filter paper activity is measured. Therefore, the results suggest that OrganoCat cellulose is more difficult to hydrolyze for the cellulolytic enzymes compared to OrganoSolv cellulose. To test this, as a next step, hydrolysis experiments have to be performed.

The experiment affirmed again that the regulation of cellulase production is very complex. It is difficult to predict the resulting filter paper activity and the overall amount of proteins produced. Furthermore, there is no clear correlation between the amount of cellulases produced and the digestibility of the substrate. Therefore, in contrast to the enzyme activity, the measurement of the respiration activity allows to estimate the amount of sugars converted. This information is most valuable to choose a suitable substrate for consolidated bioprocessing.

## Conclusions

In the current study, the respiration activity monitoring system (RAMOS) was applied to evaluate the digestibility of different celluloses by *T.* *reesei* Rut-C30 under consolidated bioprocessing conditions. The digestibility of commercial celluloses with different physicochemical characteristics was ranked using the OTR slopes and the area ratio of increasing (X) to the decreasing (Y) OTR during cellulose utilization. Analysis of the respiration activity revealed the existence of two phases during cellulose digestion, an enzyme and a cellulose-binding-sites limited phase. To test the presented method, the effects of common cellulase inducers (cellobiose and sophorose) and further substances increasing cellulase production (urea, tween 80 and peptone) were analyzed. Furthermore, miscanthus steepwater was evaluated as a new media additive showing a positive effect on the growth and cellulase production of *T. reesei.* Finally, two non-commercial cellulosic substrates, OrganoCat and OrganoSolv cellulose, were analyzed regarding their digestibility. Although nearly the same amount of cellulases was produced on both sources, OrganoSolv cellulose was easier to metabolize than OrganoCat cellulose. Surprisingly, the respiration activity on OrganoSolv cellulose was similar to that of the pure cellulosic substrate α-cellulose.

The measurement of oxygen transfer rate was established as a valuable tool to determine cellulose digestibility under fermentation conditions for CBP applications. It can be applied to evaluate and optimize lignocellulose pretreatment methods regarding their digestibility by a specific microorganism producing its particular cellulase cocktail. If the type of cellulose is fixed, the presented tool can be used to screen for the most suitable cellulose degrading microorganism. Pulse experiments using different specific enzymes would reveal the limiting enzyme activity, providing new targets for genetic improvement of the applied microorganism. Furthermore, the fermentation medium for such an application could be improved by online monitoring the effect of enzyme production enhancing compounds on cellulose digestibility.

## Methods

### Microorganism and fermentation conditions

The hypercellulolytic mutant strain *T. reesei* Rut-C30 (ATCC® 56765™) was obtained from the American Type Culture Collection (ATCC, Manassas, USA) and the spore suspension was stored at −80 °C in 20 % (v/v) glycerol. For inoculum preparation, spore suspension was sub-cultured every 4 weeks using potato extract glucose agar plates (Roth, Karlsruhe, Germany) incubated at 30 °C for 7–10 days until sporulation occurred. Afterwards, the agar plates were kept at 4 °C and harvested when needed using 10 mL 0.9 % (w/v) sodium chloride solution. The spore concentration was determined in a Neubauer-Improved counting chamber (Superior Marienfeld, Lauda-Königshofen, Germany).

All cultivations were performed in 250 mL shake flasks without baffles with a filling volume of 20 mL at 30 °C while shaking at 350 rpm with a shaking diameter of 50 mm in a Kuhner shaker ISF1-X (Kühner AG, Birsfelden, Switzerland). The culture was inoculated with a spore suspension to a final concentration of 10^6^ spores mL^−1^.

### Media and solutions

All cultures were conducted in a modified Pakula medium [50] with the following composition: (NH_4_)_2_SO_4_ 7.6 g L^−1^, KH_2_PO_4_ 2.6 g L^−1^, MgSO_4_·7H_2_O 0.5 g L^−1^, CaCl_2_·2H_2_O 0.23 g L^−1^, NaCl 0.05 g L^−1^, PIPPS 33 g L^−1^ (0.1 M), trace element solution 2.5 mL L^−1^, cellulose suspension 200 mL L^−1^. The pH-value of the medium without trace elements and cellulose was adjusted to pH 5.5 using 5 M NaOH. The trace element solution contained: citric acid 180 g L^−1^, Fe_2_(SO_4_)_3_ 2.29 g L^−1^, ZnSO_4_·7H_2_O 16 g L^−1^, CuSO_4_ 2.05 g L^−1^, MnSO_4_·7H_2_O 1.6 g L^−1^, H_3_BO_3_ 0.8 g L^−1^, CoCl_2_·6H_2_O 2.71 g L^−1^. The cellulose suspension contained 150 g L^−1^ cellulose unless otherwise specified. Additionally, the following substances were added to the medium as stock solutions at given final concertation when stated: glucose 5 g L^−1^, glycerol 5.1 g L^−1^, Celluclast^®^ 1.5L 6 FPU mL^−1^, β-glucosidase 6 IU mL^−1^, cellobiose 0.24 g L^−1^, sophorose 0.24 g L^−1^, urea 0.3 g L^−1^, peptone ex casein 2 g L^−1^ (Roth, Karlsruhe, Germany), tween 80 0.1 % (v/v). All substances except for the enzymes were diluted in water. The enzymes were diluted in 0.1 M sodium acetate solution (pH 4.8). For cellulose pulse experiments 0.2 g cellulose were added separately to each shake flask as heat–sterilized powder. All chemicals were of analytical grade and the solutions were sterile-filtered using 0.2 µm cut-off filters. Cellulose suspensions were heat sterilized at 121 °C and 1 bar overpressure for 20 min.

As commercial cellulosic substrates α-cellulose (BioReagent), Sigmacell 101, Sigmacell 20 and Sigmacell 50 purchased from Sigma-Aldrich (St. Louis, USA) were used. Their physical properties like crystallinity index (CrI) and geometric mean particle size (d_p_) are shown in Table. 1. OrganoCat cellulose was kindly provided by Philipp M. Grande (ITMC, RWTH Aachen University, Aachen, Germany). The fractionation process was performed using beech wood (particle size of 0.5–0.8 mm, loading in aqueous phase 100 g L^−1^) in a biphasic water/2-MTHF (1:1 (v/v)) system with 0.1 M oxalic acid (in total liquid volume) at 140 °C for 3 h [6]. OrganoSolv cellulose was provided by the Fraunhofer Institute for Chemical Technology (Pfinztal, Germany) prepared by using 8 % (w/w) of beech wood in a water/ethanol (35:65 (w/w)) mixture under sulfur-free conditions at 220 °C and a residence time of 3.3 h [52]. The residual lignin content of the cellulose pulp is the main impurity and was roughly estimated from literature to be 10 % (w/w) [53]. The amount of applied OrganoCat and OrganoSolv cellulose pulp was adapted to provide the same amount of carbon as with commercial celluloses.

Prior to the use of the enzymes, Celluclast® 1.5L (Novozymes, Bagsværd, Denmark) and β-glucosidase from *Aspergillus niger* (Sigma Aldrich, St. Louis, USA) were purified and rebuffered by column chromatography with an Äkta FPLC (GE Healthcare, Little Chalfont, UK) as previously described by Jäger et al. [54]. For rebuffering and subsequent dilutions 0.1 M sodium acetate solution (pH 4.8) was used.

Miscanthus steepwater was produced using 100 g L^−1^ dried miscanthus saw dust provided by Felix Winzer (INRES, Universität Bonn, Bonn, Germany) by steeping in deionized water while stirring for 4 h with a magnetic sitrrer. Subsequently, a 4-layered gauze bandage (DIN 61607-FB8, Amicus®, Germany) was used to remove the solids. To investigate effects on cellulase production, the deionized water during medium preparation was replaced by 10–100 % (v/v) with the miscanthus steepwater.

### Respiration activity monitoring system (RAMOS)

The respiration activity was monitored with an in-house constructed RAMOS. It uses oxygen partial pressure sensors and differential pressure sensors to calculate the oxygen transfer rate (OTR) and the carbon dioxide transfer rate (CTR) [30, 55]. A commercial version of the device is available from Kühner AG (Birsfelden, Switzerland) or HiTec Zang GmbH (Herzogenrath, Germany).

To evaluate cellulose conversion by the enzyme cocktail produced by *T. reesei*, several characteristic parameters of the second peak of the oxygen transfer rate were calculated. The slope of linear increase and linear decrease as well as the area ratio of increasing (X) to the decreasing (Y) OTR are marked in Fig. 4.

### Sample analytics

During cultivation, samples were taken from cotton plug-sealed shake flasks cultivated in parallel to the RAMOS flasks under identical culture conditions to avoid disruption of respiration activity measurement. The pH-value of culture broth was measured with a CyberScan pH 510 device (Eutech Instruments, The Netherlands). To determine the cellulose content, a method from Updegraff [56] adapted by Ahamed and Vermette [26] was applied. The method is based on the selective acidic hydrolysis of the fungus and gravimetric determination of the remaining cellulose content. The protein content of culture supernatant was analyzed using the Bradford assay [57] with Bradford Reagent (Sigma-Aldrich, St. Louis, USA) and bovine serum albumin as a standard, according to the manufacturer’s protocol (Thermo Scientific, Waltham, USA).

To determine glucose and cellobiose concentration in the filtered culture supernatant (0,2 µm cut-off), the samples were analyzed via HPLC (Dionex UltiMate 3000, Thermo Scientific, Waltham, USA) equipped with an organic acid-resin column (250 × 8 mm, CS-Chromatographie Service, Langerwehe, Germany) and a Shodex RI-101 detector (YMC Europe GmbH, Dinslaken, Germany). As mobile phase 5 mM H_2_SO_4_ was used at a flow rate of 0.8 mL min^−1^ and a temperature of 60 °C.

The overall cellulase activity in the culture supernatant was determined using the filter paper assay (FPA) according to Ghose [58], using the procedure of Xiao [59], who adapted the assay to a 96 µL reaction volume in microtiter plates. The assay was performed using 0.1 M sodium acetate buffer (pH 4.8) incubated in a conditioned water bath at 50 °C for 1 h. Afterwards, the reducing sugars were determined with the p-hydroxybenzoic acid hydrazide (PAHBAH) assay with glucose as a standard [60]. Each sample was measured in at least two dilutions each as triplicate. To perform the PAHBAH assay the working reagent was prepared by mixing solution A (*p*-hydroxybenoic acid hydrazide 5 g, HCl (37 % (w/w)) 5 mL ad 100 mL deionized water) and solution B (trisodium citrate 12.5 g, CaCl_2_, 1.1 g, NaOH 20 g ad 1 L deionized water) in a ratio of 1:9 directly prior to use. 100 µL working reagent was mixed with 50 µL enzyme solution after the FPA assay and incubated at 100 °C for 10 min. After cooling to room temperature, the absorbance was measured at 410 nm in a microplate reader Synergy 4 (Biotek, Winooski, USA). β-glucosidase activity was measured using the p-Nitrophenyl-β-d-glucopyranoside Assay according to Parry et al. [61]. 0.1 M sodium acetate (pH 4.8) was used as buffer.

To characterize the miscanthus steepwater, the total organic carbon amount was measured with the photometric Spectroquant TOC Cell Test 14878 (Merck, Darmstadt, Germany). To detect further unknown substances 2D-fluorescence measurements were performed in a quartz cuvette (10 × 10 mm Suprasil quartz, Hellma GmbH & Co. KG, Müllheim, Germany) using the fluorescence spectrometer Fluoromax-4 (HORIBA Jobin–Yvon GmbH, Unterhaching, Germany). A 2D-spectrum from 290 to 700 nm was measured with an excitation and emission slit set to a bandwidth of 4 nm.

## Additional files

10.1186/s12934-016-0567-7 Complete set of reference cultivations of *T. reesei* Rut-C30 generated during this work. Oxygen transfer rate (OTR) of *T.* *reesei* Rut-C30 cultivations on 5 g L^−1^ glucose and 30 g L^−1^ α-cellulose.Culture conditions: modified Pakula medium, 250 mL flask, filling volume 20 mL, shaking frequency 350 rpm, shaking diameter 50 mm, inoculum 10^6^ spores mL^−1^, and 30 °C. The lag phase was adapted as stated in the legend.
10.1186/s12934-016-0567-7 Oxygen based parameters of a *T. reesei* Rut-C30 cultivation. Slope of linear increase and decrease of oxygen transfer rate over area ratio (X/Y) in a culture of *T.* *reesei* Rut-C30 on 5 g L^−1^ glucose and 30 g L^−1^ α-cellulose as carbon sources is evaluated according Fig. 4.
10.1186/s12934-016-0567-7 Fluorescence spectrum of 100 % (v/v) miscanthus steepwater. Experimental conditions: quartz cuvette, 2 mL filling volume, spectral range of 290–700 nm with an increment of 2 nm.

## Abbreviations

CBP: consolidated bioprocessing
Crl: crystallinity index
FPA: filter paper activity
FPU: filter paper unit
OTR: oxygen transfer rate
OT: cumulative oxygen transfer
RAMOS: respiration activity monitoring system
*T. reesei*: *Trichoderma reesei*
